# Association of Exposure to Radio-Frequency Electromagnetic Field Radiation (RF-EMFR) Generated by Mobile Phone Base Stations with Glycated Hemoglobin (HbA1c) and Risk of Type 2 Diabetes Mellitus

**DOI:** 10.3390/ijerph121114519

**Published:** 2015-11-13

**Authors:** Sultan Ayoub Meo, Yazeed Alsubaie, Zaid Almubarak, Hisham Almutawa, Yazeed AlQasem, Rana Muhammed Hasanato

**Affiliations:** 1 Department of Physiology, College of Medicine, King Saud University, P.O. Box 2925, Riyadh 11461 Saudi Arabia; E-Mails: yazeed-1992-@hotmail.com (Y.A.); z-almubarak@hotmail.com (Z.A.); hisham-001@hotmail.com (H.A.); yazeed.alq@gmail.com (Y.A.); 2 Department of Clinical Bio-Chemistry, College of Medicine, King Saud University, P.O. Box 2925, Riyadh 11461 Saudi Arabia; E-Mail: rhasanato@ksu.edu.sa

**Keywords:** mobile phone radiation, mobile phone base station, RF-EMFR, HbA1c, hyperglycemia

## Abstract

Installation of mobile phone base stations in residential areas has initiated public debate about possible adverse effects on human health. This study aimed to determine the association of exposure to radio frequency electromagnetic field radiation (RF-EMFR) generated by mobile phone base stations with glycated hemoglobin (HbA1c) and occurrence of type 2 diabetes mellitus. For this study, two different elementary schools (school-1 and school-2) were selected. We recruited 159 students in total; 96 male students from school-1, with age range 12–16 years, and 63 male students with age range 12–17 years from school-2. Mobile phone base stations with towers existed about 200 m away from the school buildings. RF-EMFR was measured inside both schools. In school-1, RF-EMFR was 9.601 nW/cm^2^ at frequency of 925 MHz, and students had been exposed to RF-EMFR for a duration of 6 h daily, five days in a week. In school-2, RF-EMFR was 1.909 nW/cm^2^ at frequency of 925 MHz and students had been exposed for 6 h daily, five days in a week. 5–6 mL blood was collected from all the students and HbA1c was measured by using a Dimension Xpand Plus Integrated Chemistry System, Siemens. The mean HbA1c for the students who were exposed to high RF-EMFR was significantly higher (5.44 ± 0.22) than the mean HbA1c for the students who were exposed to low RF-EMFR (5.32 ± 0.34) (*p* = 0.007). Moreover, students who were exposed to high RF-EMFR generated by MPBS had a significantly higher risk of type 2 diabetes mellitus (*p* = 0.016) relative to their counterparts who were exposed to low RF-EMFR. It is concluded that exposure to high RF-EMFR generated by MPBS is associated with elevated levels of HbA1c and risk of type 2 diabetes mellitus.

## 1. Introduction

During the last two decades, the use of mobile phones has increased spectacularly among individuals of all age groups in both developing and developed countries. Mobile phones have become a prevalent means of communication and a part of everyday life [1]. There are about 7.3 billion mobile phone subscribers worldwide, almost equal to the world population [2]. Mobile phones are low power radio devices, transmit and receive radio frequency radiation, and are considered the strongest source of human exposure to radio frequency electromagnetic field radiation RF-EMFR. The RF-EMFR generated by mobile phone base stations ranges between 400 MHz and 3 GHz [3,4,5]. 

The extensive increase and development of new mobile phone technologies resulted in a major change of radiofrequency electromagnetic field radiation (RF-EMFR) exposure patterns in everyday settings [6,7]. To provide better services to the customers, mobile phone companies install base stations in the residential and commercial areas, including the school buildings, which stirred up widespread public concern about the hazards of RF-EMF radiation generated by mobile phone base stations (MPBS). The environment is exposed to RF-EMFR and health effects of RF-EMFR have been controversially discussed in the literature [8]. RF-EMFR can cause fatigue, headache, dizziness, tension, sleep disturbance [1], hearing and vision complaints [9]. The WHO International Agency for Research on Cancer has classified RF-EMFR as possibly carcinogen [10]. RF-EMFR promotes cancer development via stimulation of cell proliferation and apoptosis inhibition [11]. 

Presently about 382 million people are suffering from diabetes mellitus, this number is expected to upsurge to 592 million by 2035 and 183 million people are unaware of their diabetes mellitus [12]. Hemoglobin A1c (HbA1c) reflect the mean glucose concentration over the previous period of about 8–12 weeks. HbA1c is commonly used as a marker of hyperglycemia and an increased HbA1c has been regarded as an independent and reliable marker for diabetes mellitus [13]. World Health Organization, the International Diabetes Federation, and the American Diabetes Association have recently endorsed HbA1c as a diagnostic test for diabetes mellitus [13,14]. To our knowledge, this is the first study aimed to determine the association of exposure to RF-EMFR generated by MPBS with HbA1c and incidence of type 2 diabetes mellitus. 

## 2. Subjects and Methods

### 2.1. Subjects 

This cross-sectional study was conducted in the Department of Physiology, College of Medicine, King Saud University, Riyadh, Saudi Arabia. All the subjects and or their parents signed the written informed consent. The study protocol was approved by the Ethical Review Committee of College of Medicine Research Centre, King Saud University, Riyadh, Saudi Arabia (IRB-14/412).

Students were recruited based on their voluntary participation, apparently healthy status, same age, gender, nationality, regional, cultural and socio-economic status. We invited 250 participants (125 from school-1, and 125 from school-2). A detailed interview was conducted followed by clinical history taking and examination to assess whether to include in the study or not. All the students were questioned with regard to anthropometric parameters, age, height, weight, ethnicity, socioeconomic status, and family history of diabetes mellitus, blood diseases, and cigarette smoking. After clinical history and examination, finally, we selected 159 apparently healthy, male, volunteer students (96 from school-1, and 63 from School-2). The age of the students who belonged to the school-1 group was 12–16 years (mean age 13.98 ± 0.92). The age of the students who belonged to the school-2 group was 12–17 years (mean age 14.21 ± 1.99). 

### 2.2. Exclusion Criteria

Subjects with known cases of gross anemia, blood diseases, history of blood transfusion, personal or family history of known diabetes mellitus, students who suffered from marked obesity, asthma, and students who smoked tobacco were excluded from the study. Moreover, students who were living (residence) close to the any high transmission lines or MPBS and students who frequently consumed fast food and excess sweet diet were also excluded from the study. We also excluded the students who were athletes or performed regular vigorous exercise. 

### 2.3. Methods

#### 2.3.1. Selection of the Schools and Measurement of RF-EMFR 

In this study, two different elementary schools (labeled as school-1 and school-2) were selected from the Riyadh region. Both schools were located close to MPBS. It was ensured that there were no significant sources of generation and transmission of EMFR in or near the school building. In school-1 MPBS had been installed on the residential building about 200 m away from the school building. Inside the schools RF-EMF was measured by using the Narda Safety Test Solution SRM-3006. SRM-3006 is a frequency-selective field strength measurement system, which measures the RF-EMFR [15]. In this school, the RF-EMF was 9.601 nW/cm^2^ at frequency of 925 MHz, and students had been exposed to RF-EMFR for a duration of 6 h daily, five days in a week. 

The second school (school-2) was also located close to MPBS. The MPBS was installed on the residential building about 200 m away from the school building. RF-EMF was 1.909 nW/cm^2^ at frequency of 925 MHz and students had been exposed to RF-EMFR for a duration of 6 h daily, five days in a week. RF-EMFR was measured in both schools in various class rooms. We selected the points to measure RF-EMFR based on the location of class rooms. RF-EMFR was measured at three different points including the center, as well as the corners, of the class room from which we selected the students. We recorded the RF-EMFR two times per day at each point. The number of measurement was the same in the different places in the school. 

#### 2.3.2. Blood Sample Collection 

All the participants of both schools were allocated a serial number; an expert technician took 5–6 mL of blood with a vein puncture method and blood was collected in 10 mL container containing ethylenediamine tetra-acetic acid (EDTA). Blood was transferred into a container with specific code number of the student on the container. The blood was immediately kept in the refrigerator under the temperature of 4–5 °C. All blood samples were immediately transferred to the hematology laboratory, to analyze the HbA1c. 

#### 2.3.3. Measurements of HbA1c

HbA1c measurements were performed on ethylenediamine tetra-acetic acid (EDTA) blood samples, and HbA1c was measured by Dimension Xpand Plus Integrated Chemistry System, USA. The HBA1c assay on the Dimension Xpand Plus Integrated Chemistry System is an *in vitro* diagnostic assay for the quantitative determination of HBA1c in human anticoagulant whole blood. The measurement was based on the principle of turbidimetric inhibition immunoassay (TINIA). Each kit contains matched sets of HBA1c reagent cartridge and calibrators. These components were not interchangeable between the kits and other lot numbers. HBA1c required lot specific scalers which were entered before the calibration. The scaler values were provided on the reagent cartridge. The system was calibrated daily and a few samples were tested twice to check the accuracy of HbA1c with the Dimension Xpand Plus Integrated Chemistry System. 

### 2.4. Statistical Analysis

The data were computed into the computer and analyzed by using the Statistical Package for Social Sciences (SPSS for Windows, version 20.0). Unpaired Student’s t-test (parametric test with the assumption of equal variances) was applied to check the difference of the means values between the two quantitative variables. All the variables were entered into a logistic regression model and results were presented as an odds ratio and 98% confidence interval. The level of significance was assumed at *p* < 0.05. 

## 3. Results

Table 1 summarizes the comparison of the anthropometric variables and HbA1c parameters between the students of two different schools where students had been exposed to RF-EMFR generated by MPBS at 9.601 nW/cm^2^ at frequency of 925 MHz for the duration of 6 h daily, five days per week, over the last two years (school-1). While in the second school, students were exposed to RF-EMFR of 1.909 nW/cm^2^ at a frequency of 925 MHz for the duration of 6 h daily, five days per week, over the last two years (school- 2).The age of the students at school-1 (group 1) was 12–16 years (mean age 13.98 ± 0.92), while the age of the students at second school-2 (group-2) was 12–17 years (14.21 ± 1.993). 

The mean HbA1c for the students who were exposed to high RF-EMFR (9.601 nW/cm^2^ at frequency of 925 MHz) was significantly higher (5.4%) than the mean HbA1c for the students who had been exposed to low RF-EMFR (1.909 nW/cm^2^ at frequency of 925 MHz) generated by MPBS was (5.3%) (*p* = 0.007). The results show students who were exposed to high RF-EMFR have significantly impaired HbA1c (30, 31.25%) than the students who exposed to low RF-EMFR (17, 27.0%) (Table 2). It shows an association of RF-EMFR and higher risk of type 2 diabetes among the students who were exposed to high RF-EMF relative to their counterparts who were exposed to low radiation (Table 2). Logistic regression analysis showed a significant association with high RF-EMFR, HbA1c, and risk of type 2 diabetes mellitus (Table 3).

**Table 1:** Comparison of anthropometric parameters and HBA1c percentage of the students who were exposed to RF-EMFR generated by mobile phone base stations at (9.601 nW/cm^2^ at frequency of 925 MHz) *versus* the students exposed to RF-EMFR at (1.909 nW/cm^2^ at frequency of 925 MHz).

Parameters	School Group #1 (*n* = 96) RF-EMFR: 9.601 nW/cm^2^	School Group # 2 (*n* = 63) RF-EMFR: 1.909 nW/cm^2^	*p* Values
Age (years)	13.98 ± 0.92	14.21 ± 1.003	0.138
BMI (m/kg)^2^	22.91 ± 5.12	21.47 ± 5.47	0.093
HbA1c (%)	5.445 ± 0.22	5.325 ± 0.34	0.007

Note: Values are presented in mean ± SD.

**Table 2:** Comparison of prevalence of pre-diabetes mellitus based on HBA1c percentage of the students exposed to RF-EMFR generated by mobile phone base stations at (9.601 nW/cm^2^ at frequency of 925 MHz) *versus* the students exposed to RF-EMFR at (1.909 nW/cm^2^ at frequency of 925 MHz).

Parameters	School Group #1 (*n* = 96) RF-EMFR:9.601 nW/cm^2^	School Group # 2 (*n* = 63) RF-EMFR: 1.909 nW/cm^2^	*p* Values
Prevalence of Impaired HbA1c ≥5.6 (Prediabetes)	30 (31.25%)	17 (27%)	0.016

Values are presented in %. HbA1c > 5.6 was considered impaired HbA1c (pre diabetes) [16].

**Table 3:** Logistic regression analysis for variables predicting an association of RF-EMFR with HbA1c and prevalence of risk of type 2 diabetes mellitus.

Parameters	Odds Ratio	95% Confidence Interval	*p* Values
Age (years)	0.67	0.23–1.92	0.454
Obesity	1.87	0.539–6.493	0.324
Underweight	2.79	0.649–11.166	0.148
RF-EMFR	342	46–2530	0.0001

Note: The model predicts 89%.

## 4. Discussion

The findings of this study show that the students who were exposed to high RF-EMF had significantly higher HbA1c than the students who were exposed to low RF-EMF. Moreover, students who were exposed to high RF-EMFR generated by MPBS had a significantly higher proportion of diabetes mellitus relative to the students who were exposed to low RF-EMFR. 

HbA1c is well recognized among clinicians as a marker of chronic hyperglycemia, increased HbA1c has also been regarded as an independent marker for diabetes mellitus [17]. HBA1c has numerous advantages compared to the Fasting Plasma Glucose (FPG), including greater expediency, fasting is not mandatory, better pre-analytical stability and less day-to-day worries during a period of stress and illness. HbA1c has recently been endorsed as a diagnostic test for diabetes by the World Health Organization, the International Diabetes Federation, as well as the American Diabetes Association [12,14,17,18]. 

FPG of 100 mg/dL or 5.6 mmol/L equals to an HbA1c of 5.4% and FPG of 110 mg/dL or 6.1 mmol/L is parallel to HbA1C of 5.6% [13]. The normal cut-off point of HbA1c is equal to or less than 5.4%. Compared to the fasting glucose cut point of 100 mg/dL (5.6 mmol/L), the HbA1c cut point of 5.7% is more specific and has a higher positive predictive value to identify people at risk for development of diabetes. HbA1c levels below 5.7% may still be at risk to develop diabetes mellitus [13]. Literature also indicates that subjects within the HbA1C range of 5.5%–6.0% have a five-year cumulative incidence of diabetes mellitus that ranges from 12% to 25% [19]. In the present study, we found that the mean HbA1c for the students who were exposed to high RF-EMFR was 5.44% compared to the mean HbA1c for the students who were exposed to low RF-EMFR 5.32% (Table 1). 

### 4.1. RF-EMFR and HbA1c

The possibility of induction of biological and health effects by low-energy radiation emitted by MPBS remains a debatable issue. In spite of decades of research, there is still ongoing discussion about RF-EMFR and physiologically-relevant effects. Literature is available on the association of RF-EMF with headache, tension, and sleep disorder-like symptoms [1]. In addition, studies have also shown that RF-EMFR has extensive damaging effects on the nervous system, cardiovascular, and male reproductive system [20]. RF-EMFR also causes oxidative damage [21] and cancer [22].

Bieńkowski *et al.* [23] conducted a study and measured the changes in the electromagnetic field intensity in a school building and its surrounding after the MPBS installation on the roof of the school. They found that the EMF intensity increased in the building and its surroundings after the MPBS installation. Shahbazi-Gahrouei [24] conducted a cross-sectional study on people living near the mobile phone base transceiver stations (BTS). The authors reported that discomfort, irritability, nausea, headache, dizziness, nervousness, depression, sleep disturbance, memory loss, and decreased libido were statistically significant among the people living near the BTS antenna (less than 300 m distant) compared to those living far from the BTS antenna (more than 300 m). They suggested that cellular phone BTS towers should not be installed at less than a distance of 300 m to human population to minimize exposure. 

Meo *et al.* [25] determined the effects of exposure to RF-EMFR generated by mobile phones on fasting blood glucose in albino rats. The authors found that, Wister albino rats exposed to RF-EMF generated by mobile phone for more than 15 min a day for a maximum period of three months had significantly higher fasting blood glucose and serum insulin compared to the control group. Meo *et al.* [25] also reported that increase in fasting blood glucose was due to insulin resistance. In the present study, we found that students who were exposed to high RF-EMFR generated by MPBS had significantly higher HbA1c (Table 1) and a higher prevalence of type 2 diabetes mellitus (Table 2) than the students who were exposed to low RF-EMFR. 

Altpeter *et al.* [26] reported that the incidence for diabetes mellitus was higher among the subjects living within a close radius of a shortwave transmitter in Schwarzenburg, Switzerland compared with a population living away from the a shortwave transmitter. There was a significant linear relationship between RF radiation exposure and prevalence of diabetes mellitus. 

Jolley *et al.* [27] exposed the islets of Langerhans from rabbits to low-frequency pulsed magnetic fields and noted a significant decrease in insulin release during glucose stimulation compared to controls. Similarly, Sakurai *et al.* [28] measured the insulin secretion from an islet cell, exposed to low-frequency magnetic fields compared with sham exposure group. Insulin secretion was decreased by about 30% when exposed to low-frequency magnetic fields compared to sham exposure. 

Li *et al.* [29] exposed hepatocytes *in vitro* to 50 Hz pulsed EMF noted a conformation change in the insulin molecule. The authors found a decrease in the binding capacity of insulin to its receptors compared with controls. 

Congruently, Havas [30] reported that exposure to electromagnetic pollution cause higher plasma glucose levels and may contribute to diabetes mellitus. Havas [30] also concludes that decreased insulin secretion and reduced binding capacity of insulin to its receptors may explain the elevated levels of plasma glucose in subjects exposed to electromagnetic fields. Similarly, in the present study, we found that students who were exposed to high RF-EMFR generated by MPBS had significantly higher HbA1c and risk of type 2 diabetes mellitus than the students who were exposed to low RF-EMFR. Choi *et al.*, 2011 [31] reported that individuals with HbA1c ≤ 5.5 is a normal; 5.6 to 6.9 is impaired HbA1c or pre-diabetes, and HbA1c 7.0 considered as a diabetes. They also reported that HbA1c ≥5.6% have an increased risk for future diabetes. In our study, we found that students who were exposed to high RF-EMFR have significantly higher HbA1c than the mean HbA1c for the students who had been exposed to low RF-EMFR. Moreover, students exposed to high RF-EMFR have significantly impaired HbA1c (31.25%) than the students who exposed to low RF-EMFR (27.0%). 

### 4.2. What This Study Adds

The present study is one of the first studies to investigate the association of EMFR generated by MPBS with HbA1c and prevalence of type 2 diabetes mellitus. Students who were exposed to high EMFR generated by MPBS had significantly higher HbA1c and prevalence of pre diabetes mellitus compared to their students who exposed to low EMFR. We believe that EMFR appears to be another risk factor contributing to high levels of HbA1c and risk of type 2 diabetes mellitus. This notion may present a possible paradigm shift in the development of diabetes mellitus. This research provides awareness to the community and to the health officials regarding the effects of EMFR generated by MPBS on HbA1c and incidence of diabetes mellitus. 

### 4.3. Study Strengths and Limitations 

To our knowledge, no study exists yet to establish an association between the RF-EMFR generated by MPBS and HbA1c and risk of type 2 diabetes mellitus. We measured the levels of RF-EMFR inside the schools to determine the impact of RF generated by MPBS on HbA1c. In this study for subject selection criteria, we follow the American Diabetic Association guidelines, and considered age, race, ethnicity, anemia, and hemoglobinopathies into consideration while using the A1C to diagnose diabetes [32]. Moreover, our study exclusion criteria and assays are highly standardized. The limitation of the present study is the involvement of male gender only because in Saudi Arabia there is no co-education system at schools, colleges, and university levels. This study is a relatively small sample size, and because of the cross-sectional design of the study we could not establish the causation. 

## 5. Conclusions 

Exposure to high RF-EMFR generated by MPBS is associated with elevated level of HbA1c and prevalence of pre diabetes mellitus among school aged adolescents. RF-EMFR appears to be another risk factor contributing to high levels of HbA1c and incidence of type 2 diabetes mellitus. This study provides awareness to the community and to the health officials regarding the effects of RF-EMFR generated by MPBS on HbA1c and its association with type 2 diabetes mellitus. We cannot deny the services provided by the mobile phone industry but we also strongly believe that health is more important and it cannot be compromised over anything. Thus, it must be kept in mind the mobile MPBS should not be installed in the thickly populated areas, especially in or near the school buildings. 
